# Value of Liver Regeneration in Predicting Short-Term Prognosis for Patients with Hepatitis B-Related Acute-on-Chronic Liver Failure

**DOI:** 10.1155/2020/5062873

**Published:** 2020-08-06

**Authors:** Xiaoping Wang, Mengying Sun, Xianjun Yang, Liucun Gao, Min Weng, Dehui Yang, Hongyong Li, Xiaolei Zhou, Jiani Li, Sen Qin, Dejiang Zhou, Xiaoling Wu, Shanhong Tang, Weizheng Zeng

**Affiliations:** ^1^Department of Gastroenterology, The General Hospital of Western Theater Command, Chengdu, Sichuan, China 610083; ^2^Department of Gastroenterology, Suining Central Hospital, Suining, Sichuan, China 629000; ^3^College of Medicine, Southwest Jiaotong University, Chengdu, Sichuan, China 610003; ^4^Western Military Command Disease Prevention and Control Center, Chengdu, Sichuan, China 610021; ^5^Clinical Research Center, Beijing Children's Hospital, Capital Medical University, National Center for Children's Health, Beijing, China 100045

## Abstract

**Background and Aims:**

The value of hepatocyte regeneration in predicting the outcomes of hepatitis B-related acute-on-chronic liver failure (HBV-ACLF) is not fully assessed. The present study was aimed at establishing a novel scoring system to predict patients' outcomes within 3 months by applying serological indicators of hepatic regeneration and liver injury.

**Methods:**

Patients with chronic hepatitis B who had a rapid deterioration were investigated. Patients were observed for 90 days, and the endpoint of follow-up was death or liver transplantation. Serum parameters were estimated on the diagnosis of acute-on-chronic liver failure (ACLF). Cox proportional hazard regression was used to identify independent prognostic factors and create a novel prognostic scoring system, and a receiver operating characteristic (ROC) curve was used to analyze the performance of the model.

**Results:**

A total of 308 patients with HBV-ACLF were incorporated and divided into the training cohort (*n* = 206) and testing cohort (*n* = 102) randomly. Creatine (Cre), age, total bilirubin (TBil), alpha-fetoprotein (AFP), and international normalized ratio (INR) were found to be independent prognostic factors. According to the results of Cox regression analysis, a new prognostic model (we named it the TACIA score) was calculated. The areas under ROC (AUROC) for the new model were 0.861 and 0.763 in the training and testing cohorts, respectively, and patients with lower TACIA scores (<4.34) would survive longer (*P* < 0.001).

**Conclusions:**

A pertinent prognostic scoring system for patients with HBV-ACLF was established in our study, and the novel model could predict patients' short-term survival effectively.

## 1. Introduction

Acute-on-chronic liver failure is a life-threatening clinical syndrome with a rapid progress of hepatic injury on the basis of chronic liver diseases. The likely causes of acute decompensation could be either hepatic or nonhepatic. In China, hepatitis B virus (HBV) infection is a significant health concern, and the reaction of HBV becomes the most common pathogeny of ACLF. Thus, hepatitis B-related acute-on-chronic liver failure becomes a weighty problem [1]. In addition, the rapidly worsening liver dysfunction may finally result in multiple organ failure and a high short-term mortality.

Recently, liver transplantation is the most efficient method for ACLF treatment. Unfortunately, patients seldom have chance to get liver transplantation because of the severe donor liver shortage. As a result, intensive care and supportive therapy have become alternatives to manage ACLF. Except liver transplantation, currently applied therapeutic methods are aimed at helping to clear cytotoxic items and create a proper circulation for liver regeneration; for that, liver regeneration is a vital procedure to the recovery of severe hepatic injury [2]. In the past few years, the mortality of ACLF has shown a decreasing trend due to the early diagnosis and management of organ failure, but the survival rate is still not as expected [3]. Thus, prognostic models could play an essential role in ACLF management, including the Child-Pugh score (CTP) [4], MELD score [5], AARC score [6], CLIF-SOFA score [7], CLIF-C OF score, and CLIF-C ACLF score [8]. Those models mainly evaluate the severity of liver injury and the occurrence of multiple organ failure. Rarely did they focus on the capability of liver tissue repairing and liver regeneration. Functional liver tissue repairing is the key to the improvement of injured hepatic function. As a maker of liver regeneration, alpha-fetoprotein (AFP) was found to be a parameter correlated with the outcome of acute liver failure [9]. But seldom has its predictive value been assessed in ACLF. In our previous study, we found that an elevated AFP level could predict a better outcome for HBV-ACLF patients [10].

The outcome of liver failure should be assessed from the perspective of both damaged liver function and the ability of liver regeneration. But researchers mostly concentrate on the former one. Thus, we aimed to perform a timely assessment of patients' outcomes upon the diagnosis of ACLF by integrating clinical parameters of both organ damage and liver regeneration and to create and validate a new prognostic model for HBV-ACLF centering on the value of hepatic regeneration.

## 2. Patients and Methods

### 2.1. Study Cohort and Data Collection

**Figure 1:**
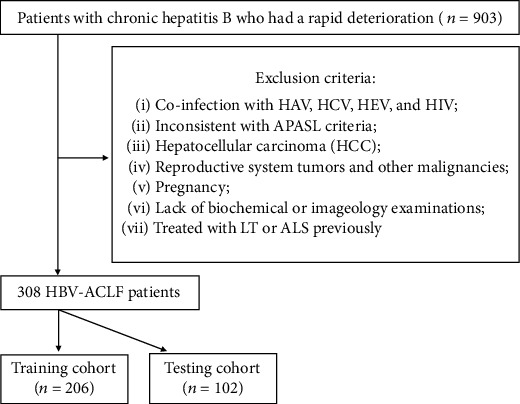
Inclusion and exclusion criteria of this research.

We retrospectively studied patients with chronic hepatitis B who have an acute progression of liver dysfunction from 2012-2-27 to 2017-9-27 in our hospital. The diagnosis of chronic hepatitis B was based on the existing guidelines. Liver cirrhosis was diagnosed by referring to liver biopsy, ultrasound, fibroscan, laboratory examination, and clinical manifestations. ACLF was diagnosed according to Asian Pacific Association for the Study of the Liver (APASL) [11]: a presentation of jaundice (serum total bilirubin ≥ 85 *μ*mol/L), coagulopathy (INR ≥ 1.5 or prothrombin activity ≤ 40%), and any degree of encephalopathy and/or clinical ascites within 4 weeks on the basis of ongoing chronic liver diseases. Patients with HBsAg positive who were aged between 18 and 80 and had a manifestation of liver dysfunction within 4 weeks were included. After preliminary screening, 903 patients with chronic hepatitis B who had an acute progress of liver dysfunction were studied. Five hundred and ninety-five patients were excluded for the following: (1) coinfection with HAV, HCV, HEV, and HIV; (2) those who do not meet the APASL criteria; (3) any evidence to hepatocellular carcinoma (HCC); (4) combination with reproductive system tumors and other malignancies; (5) pregnancy; (6) a lack of biochemical or imageology examination; and (7) those treated with liver transplantation (LT) or artificial liver support (ALS) previously. Finally, there were 308 patients incorporated into this study. The population was randomly separated into two subgroups at a proportion of 2 : 1 to establish and validate a new prognostic model (Figure 1). Clinical data was collected upon the diagnosis of ACLF. Prognostic models including CTP, MELD, AARC, CLIF-SOFA, CLIF-C OF, and CLIF-C ACLF were recorded as tools of condition assessment. Patients were followed up for 90 days since the date of ACLF diagnosis. The endpoint of follow-up is death or liver transplantation.

### 2.2. Patient Management

Standard medical treatment was obtained including bed rest, intravenous antibiotics, liver-protective treatment, and energy supplements. Patients also received plasma and albumin infusion, water-electrolyte maintenance, and complication-preventing treatment. Antiviral therapies were administered individually according to the virus replication levels and patients' conditions by using lamivudine, telbivudine, adefovir dipivoxil, or entecavir.

### 2.3. Statistical Analyses

Statistical analyses were performed by referring to SPSS software (version 16.0; IBM Corporation, Somers, NY, USA). Continuous data were expressed as means ± SD or medians with interquartile range appropriately. Those variables were compared by using Student's *t*-test or the nonparametric Mann-Whitney *U* test. Percentages were used to present categorical data, which were compared by the chi-squared test or Fisher's exact test. The independent prognostic factors were identified by multivariate Cox regression analysis, and a new prognosis scoring system was established on the basis of Cox proportional hazard regression. The area under the receiver operating characteristic curve was used for model discrimination and calibration. The comparison of cumulative survival rates was conducted with the Kaplan-Meier method. It was considered of statistical significance when *P* ≤ 0.05.

## 3. Results

### 3.1. Characteristics and Outcomes of HBV-ACLF Patients

There are 308 patients incorporated in our study.  Table 1 reveals the baseline characteristics of HBV-ACLF patients. During a 90-day follow-up, eighty-eight cases (42.72%) were deceased or got liver transplant in the training cohort, and the liver transplant-free survival rate was 53.92% (55/102) in the testing cohort. The rates of liver transplantation were 0.97% (2/206) and 4.90% (5/102) within 90 days in the training and testing cohorts, respectively.

### 3.2. Independent Prognostic Factors and Development of a New Predictive Model

In the training cohort, age (43.92 ± 11.69 years versus 52.80 ± 12.04 years, *P* < 0.001), total bilirubin (233.91 (88.50, 634.60) *μ*mol/L versus 310.60 (86.50, 795.70) *μ*mol/L, *P* = 0.003), AFP (82.19 (1.80, 3858.00) ng/mL versus 17.50 (1.04, 1155.65) ng/mL, *P* < 0.001), INR (1.81 (1.50, 4.44) versus 2.24 (1.52, 7.26), *P* < 0.001), Cre (69.20 (31.00, 207.70) *μ*mol/L versus 84.55 (39.00, 505.00) *μ*mol/L, *P* < 0.001), leukocyte count (5.82 (2.01, 25.51) × 10^9^/L versus 7.37 (1.80, 37.50) × 10^9^/L, *P* < 0.001), and albumin (31.71 (18.60, 43.90) g/L versus 30.48 (13.60, 40.60) g/L, *P* = 0.003) are of statistical significance in survivors and patients with poor outcomes.

After univariate Cox regression, clinically significant parameters were verified by multivariate analysis. Finally, total bilirubin, creatine, age, INR, and AFP were found to be independent factors of patients' outcomes (Table 2). Then, a new prognostic model (we named it the TACIA score) for HBV-ACLF patients was established as the following mathematical formula: TACIA score = 0.003 × TBil (*μ*mol/L) + 0.036 × age + 0.009 × Cre (*μ*mol/L) + 0.525 × INR–0.003 × AFP (ng/mL).

### 3.3. Performance of the New Model

**Figure 2:**
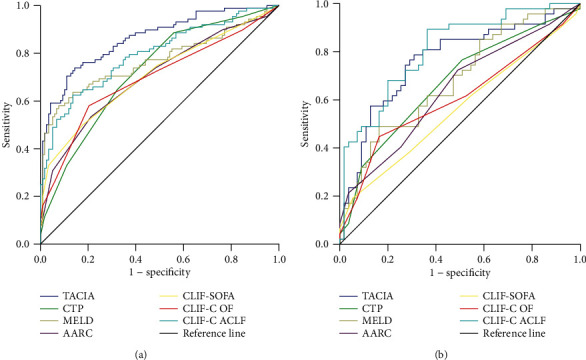
The performance of the novel scoring system compared with that of other models: (a) training cohort; (b) testing cohort.

Firstly, the performance of the TACIA score was estimated internally in the training cohort (Figure 2(a)), and its area under the ROC curve was 0.861. In addition, we compared the efficiency of the TACIA score and other formulas (including CTP, MELD, CLIF-SOFA, CLIF-C OF, and CLIF-C ACLF scores) in predicting short-term prognosis. The results illustrated that the TACIA score was superior to those models mentioned above. Furthermore, we externally examined the performance of the novel predictive model in the testing cohort (Figure 2(b)), and it showed its validity as well (AUROC = 0.763). The areas under the ROC curve of each model were compared with TACIA by the *z* test in both the training and testing cohorts.  Table 3 demonstrates the differences between TACIA and other models.

**Figure 3:**
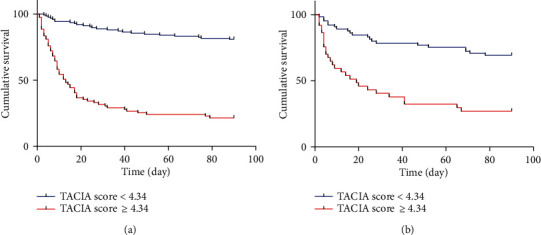
Survival curve of HBV-ACLF patients: (a) training cohort; (b) testing cohort.

The newly founded TACIA score showed its applicability in predicting a poor prognosis within 90 days in the training cohort. A cut‐off point of the TACIA score ≥ 4.34 was suggested to indicate a poor outcome with 72.73% sensitivity and 86.44% specificity. The results demonstrated that patients with a higher TACIA score (≥4.34) would have increased risk for poor outcomes. Thus, we further analyzed patients' survival according to their TACIA scores (Figure 3). In the training cohort, the transplant-free survival rate at 28 and 90 days were 32.10% (26/81) versus 88.80% (111/125) (*P* < 0.001) and 20.99% (17/81) versus 80.80% (101/125) (*P* < 0.001) in groups of patients with TACIA score ≥ 4.34 and <4.34. In the testing cohort, the transplant-free survival rates at 28 and 90 days were 44.24% (16/37) versus 80.00% (52/65) (*P* < 0.001) and 27.03% (10/37) versus 69.23% (45/65) (*P* < 0.001), respectively.

## 4. Discussion

Acute-on-chronic liver failure is a serious clinical syndrome that exhibits a high short-term mortality. Effective prognostic models could be of great value in the management of ACLF and predicting patients' outcomes, including CTP, MELD, and other prognostic formulas. Previous studies have illustrated that these formulas could be efficient tools to estimate the prognosis of cirrhosis and end-stage liver diseases, but their efficiencies might vary from territories and etiologies. In addition, these models mainly assess the condition of organ failure, so that they may not be inadequate enough to evaluate the prognosis of HBV-ACLF. Except for the severity of organ failure, the capability of hepatic regeneration could also be an essential item to the prognosis of ACLF. It is acknowledged that the liver shows its tissue repairing potential after hepatic resection or obvious hepatocyte necrosis. So, liver regeneration could be a significant procedure to the reversal of impaired hepatic function.

Liver transplantation is the most effective therapy for ACLF patients at present, while the lack of a donor liver has made it difficult and even impossible for clinical dissemination. Alternative methods including intensive care and antiviral therapy could help control the progression of liver dysfunction and promote hepatic repairing. The secretion of AFP is minimal in an adult liver, and it could usually be detected just in pathophysiological situations including hepatocyte proliferation and canceration [12]. As a marker of liver regeneration, AFP could be a prognostic item for patients with liver damage [13]. Previous researches have expounded that elevated AFP levels could predict a better prognosis for acute liver failure [14, 15]. Yet its prognostic value in ACLF has not been fully clarified. Considering the predictive value of liver regeneration, a formula combining AFP with other indices of liver function to estimate the prognosis of HBV-ACLF should be proposed.

The present study illustrated that total bilirubin, age, creatine, INR, and AFP were independent factors of patients' outcomes. The level of bilirubin would be elevated when massive necrosis of hepatocytes occurred or under the conditions of biliary obstruction and hemolysis. Patients with severe liver damage exhibit diminished liver function which may lead to multiple organ dysfunction and high short-term mortality. High serum bilirubin concentrations in ACLF patients could indicate an apparent injury of hepatocytes, which was always associated with poor outcomes [16]. Besides, there is a growing risk of poor outcomes for HBV-ACLF patients along with the increase in serum creatine. Patients with a higher creatine level could carry a kidney dysfunction and even to the extent hepatorenal syndrome, which may finally result in unexpected outcomes [17, 18]. Consistent with Cordoba et al.'s research, we found that INR was an independent risk factor of short-term mortality for ACLF patients [19]. The liver would have a weakened synthesis of coagulation factors when got severely injured, which may lead to coagulopathy and even multiorgan failure. Except for the severity of liver damage, the ability of hepatic regeneration is also a key to the prognosis of liver failure. AFP is considered a marker of hepatocyte regeneration in liver injury and could predict patients' outcomes [20]. Besides, elderly patients with chronic hepatitis B are at risk of developing HBV-ACLF, and age is of prognostic significance. Studies illustrated that elderly HBV-ACLF patients tend to have a higher 3-month mortality [21] [22]. Those patients would exhibit a declined systemic health condition, and the capability of liver regeneration may be diminished, so that the prognosis sometimes trends to be unexpected [23, 24].

The novel scoring system showed its prognostic value for HBV-ACLF by calculating age, creatine, INR, and AFP. The new model could predict the 90-day survival effectively and has an advantage over CTP, MELD, CLIF-SOFA, CLIF-C OF, and CLIF-C ACLF in the training cohort. The novel scoring system also showed its applicability in the testing cohort though no significantly statistical difference was found between TACIA and CTP, MELD, and CLIF-C ACLF, for which the limited sample size might be a potential reason. Patients who have lower TACIA scores (<4.34) might survive longer than those who have a higher TACIA score (≥4.34). The results indicate that patients with high TACIA scores might have a serious liver dysfunction and even incorporated with multiorgan failure, and the capability of hepatic regeneration would be diminished.

Acute-on-chronic liver failure is a syndrome featured as having poor short-term prognosis. This study highlighted a timely assessment of organ dysfunction and liver regeneration at the development of ACLF, which could help in patients' management. However, there exist some limitations in this study. Firstly, the model was constructed by the baseline clinical characteristics; for that, a dynamic observation of serological indicators was lacking. Besides, there is a shortage of multicentre comparative analysis in this research. Hence, further large-scale multicentre prospective studies assessing the availability of this novel prognostic model should be recommended.

## 5. Conclusion

In summary, the novel model could predict the prognosis of HBV-ACLF effectively. Lower levels of this new model could indicate a better outcome. The results of our research might be helpful in the management of HBV-ACLF for clinicians.

## Figures and Tables

**Table 1:** Clinical characteristics and outcomes of HBV-ACLF patients.

	Training cohort (*n* = 206)	Testing cohort (*n* = 102)	*P*
Age (years)	47.71 ± 12.60	47.38 ± 11.77	0.825
Gender (male, %)	174 (84.47%)	82 (80.39%)	0.369
TBil (*μ*mol/L)	252.55 (86.50, 795.70)	281.86 (86.61, 1004.50)	0.528
Cre (*μ*mol/L)	73.70 (31.00, 505.00)	74.30 (43.00, 371.00)	0.793
Alb (g/L)	30.90 ± 4.97	31.39 ± 5.33	0.428
Leukocyte count (×10^9^/L)	6.60 (1.80, 37.50)	6.42 (2.16, 21.60)	0.997
Neutrophil count (×10^9^/L)	4.31 (0.64, 33.38)	4.54 (0.86, 16.83)	0.778
ALT (IU/L)	365.85 (21.80, 5124.20)	302.00 (16.20, 6189.40)	0.988
AST (IU/L)	302.25 (40.50, 7025.20)	318.35 (49.90, 3562.70)	0.883
INR	1.95 (1.50, 7.26)	1.91 (1.50, 6.96)	0.440
AFP (ng/mL)	52.75 (1.04, 3858.00)	35.87 (0.83, 1495.82)	0.740
HBeAg positive (%)	47 (22.82%)	29 (28.43%)	0.282
HBV DNA (log_10_ IU/mL)	5.07 (2.01, 9.76)	5.49 (2.30, 9.81)	0.298
HE (%)	109 (52.91%)	55 (53.92%)	0.867
Ascites (%)	142 (68.93%)	79 (77.45%)	0.118
28-day mortality (%)	67 (32.52%)	32 (31.37%)	0.839
90-day mortality (%)	86 (41.75%)	42 (41.18%)	0.924

AFP: alpha-fetoprotein; Alb: albumin; ALT: alanine aminotransferase; AST: aspartate aminotransferase; Cre: creatine; HE: hepatic encephalopathy; INR: international normalized ratio; TBil: total bilirubin.

**Table 2:** Univariate and multivariate Cox regression analyses of 90-day mortality in the training cohort.

	Univariate analyses			Multivariate analyses		
	*β*	HR (95% CI)	*P*	*β*	HR (95% CI)	*P*
Age (years)	0.042	1.043 (1.026, 1.061)	<0.001	0.036	1.037 (1.017, 1.056)	<0.001
Gender (male)	-0.045	0.956 (0.540, 1.693)	0.878			
TBil (*μ*mol/L)	0.003	1.003 (1.001, 1.004)	<0.001	0.003	1.003 (1.001, 1.005)	<0.001
Cre (*μ*mol/L)	0.010	1.010 (1.008, 1.013)	<0.001	0.008	1.008 (1.004, 1.011)	<0.001
Alb (g/L)	-0.074	0.929 (0.891, 0.968)	0.001	-0.010	0.990 (0.939, 1.045)	0.728
Leukocyte count (×10^9^/L)	0.110	1.116 (1.076, 1.157)	<0.001	-0.114	0.892 (0.742, 1.074)	0.227
Neutrophil count (×10^9^/L)	0.127	1.136 (1.095, 1.178)	<0.001	0.169	1.184 (0.965, 1.454)	0.105
ALT (IU/L)	0.000	1.000 (0.999, 1.000)	0.053			
AST (IU/L)	0.000	1.000 (1.000, 1.000)	0.672			
HBeAg positive (%)	0.115	1.122 (0.675, 1.864)	0.657			
HBV DNA (log_10_ IU/mL)	-0.045	0.956 (0.860, 1.064)	0.956			
INR	0.775	2.170 (1.802, 2.612)	<0.001	0.525	1.691 (1.333, 2.146)	<0.001
AFP (ng/mL)	-0.004	0.996 (0.994, 0.998)	0.001	-0.003	0.997 (0.995, 0.999)	0.021
HE	-0.163	0.849 (0.559, 1.291)	0.445			
Ascites	0.786	2.194 (1.291, 3.727)	0.004	0.307	1.360 (0.739, 2.502)	0.323

AFP: alpha-fetoprotein; Alb: albumin; ALT: alanine aminotransferase; AST: aspartate aminotransferase; Cre: creatine; HE: hepatic encephalopathy; INR: international normalized ratio; TBil: total bilirubin; HR: hazard ratio; CI: confidence interval.

**Table 3:** Performance of those prognostic models in the training and testing cohorts.

	Training cohort			Testing cohort		
	AUROC	95% CI	*P*	AUROC	95% CI	*P*
TACIA	0.861	(0.806, 0.905)		0.763	(0.669, 0.842)	
CTP	0.722	(0.655, 0.782)	<0.001	0.670	(0.570, 0.760)	0.176
MELD	0.768	(0.704, 0.824)	0.001	0.680	(0.580, 0.769)	0.076
AARC	0.701	(0.633, 0.762)	<0.001	0.641	(0.540, 0.733)	0.020
CLIF SOFA	0.707	(0.640, 0.768)	<0.001	0.562	(0.460, 0.660)	0.001
CLIF-C OF	0.695	(0.627, 0.759)	<0.001	0.607	(0.505, 0.702)	0.012
CLIF-C ACLF	0.793	(0.731, 0.846)	0.002	0.808	(0.718, 0.879)	0.308

## Data Availability

All data arising from this study are contained within the manuscript.
